# 1-{5-[4-(Hex­yloxy)phen­yl]-3-phenyl-4,5-dihydro-1*H*-pyrazol-1-yl}ethanone

**DOI:** 10.1107/S1600536810033970

**Published:** 2010-09-04

**Authors:** Asghar Abbas, Safdar Hussain, Noureen Hafeez, Kong Mun Lo, Aurangzeb Hasan

**Affiliations:** a Department of Chemistry, Quaid-i-Azam University, Islamabad 45320, Pakistan; b Department of Forensic Medicine & Toxicology, National University of Sciences & Technology, Islamabad, Pakistan; c Department of Chemistry, University of Malaya, 50603 Kuala Lumpur, Malaysia

## Abstract

The crystal structure of the title compound, C_23_H_28_N_2_O_2_, is composed of discrete mol­ecules with bond lengths and angles quite typical for pyrazoline derivatives of this class. The plane containing the pyrazoline unit is nearly planar with the mean plane of the phenyl ring at the 3-position, making a dihedral angle of 1.96 (3)°. The crystal packing is stabilized by weak C—H⋯π inter­actions involving both of the aromatic rings.

## Related literature

For the biological activity and pharmacological properties of 2-pyrazoline derivatives, see: Cottineau *et al.* (2002 ▶); Dhal *et al.* (1975 ▶); Regaila *et al.* (1979 ▶); Rathish *et al.* (2009 ▶); Subbaramaiah *et al.* (2002 ▶); Manna *et al.* (2002 ▶). For the syntheses and crystal structures of 2-pyrazoline derivatives, see: Bai *et al.* (2009 ▶); Lu *et al.* (2008 ▶); Fahrni *et al.* (2003 ▶); Jian *et al.* (2008 ▶).
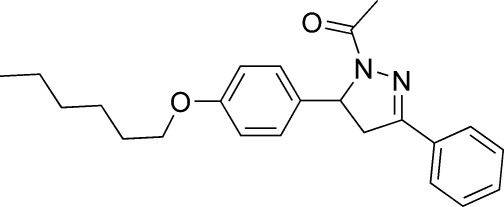


## Experimental

### 

#### Crystal data

C_23_H_28_N_2_O_2_
*M* *_r_* = 364.47
Monoclinic, 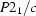

*a* = 5.3937 (8) Å
*b* = 20.237 (3) Å
*c* = 18.163 (3) Å
β = 95.144 (2)°
*V* = 1974.6 (5) Å^3^
*Z* = 4
Mo *K*α radiation
μ = 0.08 mm^−1^
*T* = 100 K
0.3 × 0.2 × 0.18 mm

#### Data collection

Bruker APEXII CCD area-detector diffractometer
Absorption correction: multi-scan (*SADABS*; Sheldrick, 1996 ▶) *T*_min_ = 0.675, *T*_max_ = 0.746
18731 measured reflections
4517 independent reflections
3430 reflections with *I* > 2σ(*I*)
*R*_int_ = 0.042

#### Refinement

*R*[*F* ^2^ > 2σ(*F* ^2^)] = 0.041
*wR*(*F* ^2^) = 0.103
*S* = 1.03
4517 reflections
246 parameters
H-atom parameters constrained
Δρ_max_ = 0.28 e Å^−3^
Δρ_min_ = −0.21 e Å^−3^

### 

Data collection: *APEX2* (Bruker, 2008 ▶); cell refinement: *SAINT* (Bruker, 2008 ▶); data reduction: *SAINT*; program(s) used to solve structure: *SHELXS97* (Sheldrick, 2008 ▶); program(s) used to refine structure: *SHELXL97* (Sheldrick, 2008 ▶); molecular graphics: *ORTEP-3* (Farrugia, 1997 ▶); software used to prepare material for publication: *publCIF* (Westrip, 2010 ▶).

## Supplementary Material

Crystal structure: contains datablocks I, global. DOI: 10.1107/S1600536810033970/zq2055sup1.cif

Structure factors: contains datablocks I. DOI: 10.1107/S1600536810033970/zq2055Isup2.hkl

Additional supplementary materials:  crystallographic information; 3D view; checkCIF report

## Figures and Tables

**Table 1:** Hydrogen-bond geometry (Å, °) *Cg1* and *Cg2* are the centroids of the C7–C12 and C16–C21 aromatic rings, respectively.

*D*—H⋯*A*	*D*—H	H⋯*A*	*D*⋯*A*	*D*—H⋯*A*
C8—H8⋯*Cg*2^i^	0.93	2.95	3.6252 (15)	131
C14—H14*A*⋯*Cg*2^ii^	0.97	2.63	3.5024 (14)	151
C19—H19⋯*Cg*1^iii^	0.93	2.71	3.4299 (15)	135

Symmetry codes: (i) 
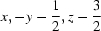
; (ii) 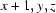; (iii) 
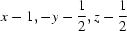
.

**Fig. 1.**
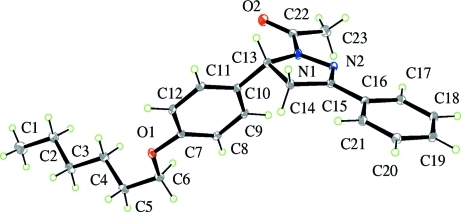
Molecular structure of (I) showing atom numbering scheme. Displacement ellipsoids are drawn at the 50% probability level.

## supplementary crystallographic information

### Comment

Pyrazoline systems are well known nitrogen-containing heterocyclic compounds which exhibit a wide range of biological activities and pharmacological properties such as anti-hyperglycemic (Cottineau *et al.*, 2002), antifungal (Dhal *et al.*, 1975), anti-diabetic, anaesthetic and analgesic properties (Regaila *et al.*, 1979), anti-inflammation (Rathish *et al.*, 2009), anticancer (Subbaramaiah *et al.*, 2002), and monoamine oxidases inhibitors (Manna *et al.*, 2002).

The molecular structure of the title compound is shown in Fig. 1. The asymmetric unit consists of discrete [PhCOCH_3_C_6_H_4_C_3_H_3_N_2_O(CH_2_)_5_CH_3_] entities, devoid of any classical hydrogen bonds. All bond lengths and angles are in the normal range (Bai *et al.*, 2009; Lu *et al.*, 2008). In the pyrazolinyl ring, the C—N and C=N bond lengths of 1.4753 (17) and 1.2856 (17) are comparable with those in similar structures [C—N 1.482 (2)–1.515 (9) Å, C=N 1.291 (2)–1.300 (10) Å] (Fahrni *et al.*, 2003). The N—N bond length of 1.3853 (15) is longer than  in the structure of Jian *et al.* [N–N 1.373 (2)–1.380 (8) Å]. The plane containing the pyrazoline moiety is nearly planar with the mean plane of the phenyl ring C16–C21 making a dihedral angle of 1.96 (3)°.

The crystal packing is stabilized by weak C-H···π interactions involving both of the phenyl rings.

### Experimental

A mixture of *(E)*-3-(4-(hexyloxy)phenyl)-1-phenylprop-2-en-1-one (3.08 g, 10 mmol) and hydrazine hydrate (1.0 g, 20 mmol) was taken in acetic acid (25 ml), and two drops of concentrated hydrochloric acid were added. The mixture was refluxed for 6 h. The precipitated solids were filtered, dried and recrystallized from ethanol. The single crystals were obtained from a mixture of ethyl acetate and dichloromethane by slow evaporation.

### Refinement

All hydrogen atoms were placed in calculated positions as riding on their parent carbon atoms with C–H = 0.93 to 0.97 Å and with *U*_iso_(H) set to 1.2 or 1.5 times *U*_eq_(C).

### Crystal data

**Table:**

C_23_H_28_N_2_O_2_	*F*(000) = 784
*M**_r_* = 364.47	*D*_x_ = 1.226 Mg m^−^^3^
Monoclinic, *P*2_1_/*c*	Mo *K*α radiation, λ = 0.71073 Å
Hall symbol: -P 2ybc	Cell parameters from 3895 reflections
*a* = 5.3937 (8) Å	θ = 2.3–28.1°
*b* = 20.237 (3) Å	µ = 0.08 mm^−^^1^
*c* = 18.163 (3) Å	*T* = 100 K
β = 95.144 (2)°	Block, white
*V* = 1974.6 (5) Å^3^	0.3 × 0.2 × 0.18 mm
*Z* = 4	

### Data collection

**Table:**

Bruker APEXII CCD area-detector diffractometer	4517 independent reflections
Radiation source: fine-focus sealed tube	3430 reflections with *I* > 2σ(*I*)
graphite	*R*_int_ = 0.042
ω scans	θ_max_ = 27.5°, θ_min_ = 1.5°
Absorption correction: multi-scan (*SADABS*; Sheldrick, 1996)	*h* = −7→6
*T*_min_ = 0.675, *T*_max_ = 0.746	*k* = −25→26
18731 measured reflections	*l* = −23→23

### Refinement

**Table:**

Refinement on *F*^2^	Primary atom site location: structure-invariant direct methods
Least-squares matrix: full	Secondary atom site location: difference Fourier map
*R*[*F*^2^ > 2σ(*F*^2^)] = 0.041	Hydrogen site location: inferred from neighbouring sites
*wR*(*F*^2^) = 0.103	H-atom parameters constrained
*S* = 1.03	*w* = 1/[σ^2^(*F*_o_^2^) + (0.0466*P*)^2^ + 0.4272*P*] where *P* = (*F*_o_^2^ + 2*F*_c_^2^)/3
4517 reflections	(Δ/σ)_max_ < 0.001
246 parameters	Δρ_max_ = 0.28 e Å^−^^3^
0 restraints	Δρ_min_ = −0.21 e Å^−^^3^

### Special details

**Table:**

Geometry. All e.s.d.'s (except the e.s.d. in the dihedral angle between two l.s. planes) are estimated using the full covariance matrix. The cell e.s.d.'s are taken into account individually in the estimation of e.s.d.'s in distances, angles and torsion angles; correlations between e.s.d.'s in cell parameters are only used when they are defined by crystal symmetry. An approximate (isotropic) treatment of cell e.s.d.'s is used for estimating e.s.d.'s involving l.s. planes.
Refinement. Refinement of *F*^2^ against ALL reflections. The weighted *R*-factor *wR* and goodness of fit *S* are based on *F*^2^, conventional *R*-factors *R* are based on *F*, with *F* set to zero for negative *F*^2^. The threshold expression of *F*^2^ > σ(*F*^2^) is used only for calculating *R*-factors(gt) *etc*. and is not relevant to the choice of reflections for refinement. *R*-factors based on *F*^2^ are statistically about twice as large as those based on *F*, and *R*- factors based on ALL data will be even larger.

### Fractional atomic coordinates and isotropic or equivalent isotropic displacement parameters (Å^2^)

**Table:**

	*x*	*y*	*z*	*U*_iso_*/*U*_eq_	
O1	0.18742 (16)	0.45980 (5)	−0.05993 (5)	0.0179 (2)	
O2	0.32678 (19)	0.17097 (5)	0.05044 (5)	0.0271 (2)	
N1	0.1119 (2)	0.20218 (6)	0.14456 (6)	0.0191 (3)	
N2	−0.0638 (2)	0.18796 (6)	0.19341 (6)	0.0179 (2)	
C1	0.5550 (4)	0.54544 (9)	−0.39381 (9)	0.0418 (4)	
H1A	0.4064	0.5562	−0.4245	0.063*	
H1B	0.6700	0.5234	−0.4229	0.063*	
H1C	0.6297	0.5852	−0.3734	0.063*	
C2	0.4902 (3)	0.50039 (7)	−0.33153 (7)	0.0249 (3)	
H2A	0.6419	0.4891	−0.3014	0.030*	
H2B	0.4195	0.4598	−0.3526	0.030*	
C3	0.3071 (3)	0.53097 (7)	−0.28225 (7)	0.0249 (3)	
H3A	0.3774	0.5716	−0.2613	0.030*	
H3B	0.1551	0.5421	−0.3123	0.030*	
C4	0.2433 (3)	0.48570 (7)	−0.21971 (7)	0.0225 (3)	
H4A	0.3943	0.4769	−0.1881	0.027*	
H4B	0.1845	0.4439	−0.2408	0.027*	
C5	0.0473 (3)	0.51279 (7)	−0.17220 (7)	0.0209 (3)	
H5A	0.1100	0.5529	−0.1480	0.025*	
H5B	−0.1010	0.5241	−0.2039	0.025*	
C6	−0.0217 (2)	0.46428 (7)	−0.11418 (7)	0.0187 (3)	
H6A	−0.1675	0.4796	−0.0916	0.022*	
H6B	−0.0584	0.4214	−0.1363	0.022*	
C7	0.1846 (2)	0.41158 (6)	−0.00717 (6)	0.0151 (3)	
C8	−0.0066 (2)	0.36643 (7)	−0.00220 (7)	0.0165 (3)	
H8	−0.1487	0.3685	−0.0351	0.020*	
C9	0.0156 (2)	0.31812 (7)	0.05236 (7)	0.0167 (3)	
H9	−0.1132	0.2880	0.0558	0.020*	
C10	0.2267 (2)	0.31401 (6)	0.10170 (6)	0.0152 (3)	
C11	0.4149 (2)	0.36037 (7)	0.09649 (7)	0.0168 (3)	
H11	0.5562	0.3586	0.1298	0.020*	
C12	0.3957 (2)	0.40882 (7)	0.04292 (7)	0.0165 (3)	
H12	0.5230	0.4395	0.0402	0.020*	
C13	0.2542 (2)	0.26318 (7)	0.16271 (7)	0.0173 (3)	
H13	0.4306	0.2524	0.1742	0.021*	
C14	0.1407 (2)	0.28567 (7)	0.23384 (7)	0.0174 (3)	
H14A	0.2658	0.2871	0.2756	0.021*	
H14B	0.0634	0.3288	0.2274	0.021*	
C15	−0.0501 (2)	0.23286 (7)	0.24369 (7)	0.0160 (3)	
C16	−0.2154 (2)	0.23144 (7)	0.30378 (7)	0.0163 (3)	
C17	−0.3931 (2)	0.18129 (7)	0.30687 (7)	0.0177 (3)	
H17	−0.4052	0.1482	0.2712	0.021*	
C18	−0.5505 (2)	0.18075 (7)	0.36271 (7)	0.0194 (3)	
H18	−0.6679	0.1473	0.3644	0.023*	
C19	−0.5348 (2)	0.22987 (7)	0.41648 (7)	0.0193 (3)	
H19	−0.6420	0.2295	0.4538	0.023*	
C21	−0.1990 (2)	0.28018 (7)	0.35816 (7)	0.0193 (3)	
H21	−0.0806	0.3135	0.3571	0.023*	
C20	−0.3591 (3)	0.27924 (7)	0.41409 (7)	0.0199 (3)	
H20	−0.3478	0.3121	0.4501	0.024*	
C22	0.1542 (3)	0.16031 (7)	0.08809 (7)	0.0209 (3)	
C23	−0.0247 (3)	0.10362 (7)	0.07542 (8)	0.0259 (3)	
H23A	0.0172	0.0784	0.0335	0.039*	
H23B	−0.1911	0.1204	0.0662	0.039*	
H23C	−0.0145	0.0758	0.1184	0.039*	

### Atomic displacement parameters (Å^2^)

**Table:**

	*U*^11^	*U*^22^	*U*^33^	*U*^12^	*U*^13^	*U*^23^
O1	0.0188 (5)	0.0165 (5)	0.0177 (4)	−0.0025 (4)	−0.0017 (3)	0.0030 (4)
O2	0.0332 (6)	0.0255 (6)	0.0248 (5)	0.0035 (5)	0.0155 (4)	0.0011 (4)
N1	0.0223 (6)	0.0178 (6)	0.0185 (5)	−0.0017 (5)	0.0093 (4)	0.0004 (4)
N2	0.0189 (6)	0.0197 (6)	0.0160 (5)	0.0009 (5)	0.0067 (4)	0.0018 (4)
C1	0.0560 (12)	0.0388 (11)	0.0334 (8)	0.0093 (9)	0.0187 (8)	0.0104 (8)
C2	0.0299 (8)	0.0235 (8)	0.0209 (7)	−0.0010 (6)	0.0005 (6)	0.0014 (6)
C3	0.0329 (8)	0.0212 (8)	0.0205 (6)	−0.0001 (6)	0.0010 (6)	0.0028 (6)
C4	0.0271 (8)	0.0209 (8)	0.0194 (6)	0.0007 (6)	0.0005 (5)	0.0018 (5)
C5	0.0247 (7)	0.0179 (7)	0.0191 (6)	0.0021 (6)	−0.0026 (5)	0.0009 (5)
C6	0.0179 (7)	0.0187 (7)	0.0187 (6)	0.0010 (5)	−0.0024 (5)	−0.0003 (5)
C7	0.0168 (6)	0.0146 (7)	0.0145 (6)	0.0021 (5)	0.0038 (5)	−0.0007 (5)
C8	0.0138 (6)	0.0193 (7)	0.0163 (6)	0.0009 (5)	0.0021 (5)	−0.0024 (5)
C9	0.0147 (6)	0.0187 (7)	0.0175 (6)	−0.0023 (5)	0.0060 (5)	−0.0016 (5)
C10	0.0163 (6)	0.0156 (7)	0.0145 (6)	0.0012 (5)	0.0061 (5)	−0.0005 (5)
C11	0.0145 (6)	0.0217 (7)	0.0144 (6)	0.0013 (5)	0.0023 (5)	−0.0015 (5)
C12	0.0142 (6)	0.0179 (7)	0.0179 (6)	−0.0027 (5)	0.0041 (5)	−0.0012 (5)
C13	0.0167 (7)	0.0189 (7)	0.0168 (6)	0.0010 (5)	0.0040 (5)	0.0015 (5)
C14	0.0186 (7)	0.0184 (7)	0.0156 (6)	−0.0004 (5)	0.0041 (5)	0.0017 (5)
C15	0.0155 (6)	0.0169 (7)	0.0159 (6)	0.0022 (5)	0.0017 (5)	0.0028 (5)
C16	0.0158 (6)	0.0190 (7)	0.0144 (6)	0.0026 (5)	0.0022 (5)	0.0031 (5)
C17	0.0188 (7)	0.0178 (7)	0.0165 (6)	0.0014 (5)	0.0012 (5)	−0.0006 (5)
C18	0.0190 (7)	0.0207 (7)	0.0190 (6)	−0.0014 (6)	0.0036 (5)	0.0035 (5)
C19	0.0185 (7)	0.0240 (8)	0.0160 (6)	0.0039 (6)	0.0057 (5)	0.0042 (5)
C21	0.0191 (7)	0.0188 (7)	0.0201 (6)	−0.0018 (6)	0.0026 (5)	0.0013 (5)
C20	0.0241 (7)	0.0205 (7)	0.0154 (6)	0.0021 (6)	0.0038 (5)	−0.0006 (5)
C22	0.0270 (8)	0.0194 (8)	0.0172 (6)	0.0053 (6)	0.0066 (5)	0.0024 (5)
C23	0.0345 (8)	0.0225 (8)	0.0214 (7)	0.0005 (6)	0.0066 (6)	−0.0027 (6)

### Geometric parameters (Å, °)

**Table:**

O1—C7	1.3688 (15)	C9—C10	1.3867 (17)
O1—C6	1.4325 (14)	C9—H9	0.9300
O2—C22	1.2226 (17)	C10—C11	1.3918 (18)
N1—C22	1.3652 (17)	C10—C13	1.5094 (17)
N1—N2	1.3853 (15)	C11—C12	1.3786 (18)
N1—C13	1.4753 (17)	C11—H11	0.9300
N2—C15	1.2856 (17)	C12—H12	0.9300
C1—C2	1.518 (2)	C13—C14	1.5471 (17)
C1—H1A	0.9600	C13—H13	0.9800
C1—H1B	0.9600	C14—C15	1.5054 (18)
C1—H1C	0.9600	C14—H14A	0.9700
C2—C3	1.523 (2)	C14—H14B	0.9700
C2—H2A	0.9700	C15—C16	1.4701 (17)
C2—H2B	0.9700	C16—C21	1.3932 (18)
C3—C4	1.5225 (19)	C16—C17	1.4008 (19)
C3—H3A	0.9700	C17—C18	1.3795 (18)
C3—H3B	0.9700	C17—H17	0.9300
C4—C5	1.525 (2)	C18—C19	1.3906 (19)
C4—H4A	0.9700	C18—H18	0.9300
C4—H4B	0.9700	C19—C20	1.380 (2)
C5—C6	1.5109 (19)	C19—H19	0.9300
C5—H5A	0.9700	C21—C20	1.3912 (18)
C5—H5B	0.9700	C21—H21	0.9300
C6—H6A	0.9700	C20—H20	0.9300
C6—H6B	0.9700	C22—C23	1.504 (2)
C7—C8	1.3868 (18)	C23—H23A	0.9600
C7—C12	1.3934 (17)	C23—H23B	0.9600
C8—C9	1.3896 (18)	C23—H23C	0.9600
C8—H8	0.9300		
			
C7—O1—C6	117.98 (10)	C9—C10—C13	122.44 (12)
C22—N1—N2	121.49 (11)	C11—C10—C13	119.01 (11)
C22—N1—C13	124.64 (11)	C12—C11—C10	121.22 (12)
N2—N1—C13	113.75 (10)	C12—C11—H11	119.4
C15—N2—N1	108.01 (11)	C10—C11—H11	119.4
C2—C1—H1A	109.5	C11—C12—C7	119.64 (12)
C2—C1—H1B	109.5	C11—C12—H12	120.2
H1A—C1—H1B	109.5	C7—C12—H12	120.2
C2—C1—H1C	109.5	N1—C13—C10	113.03 (10)
H1A—C1—H1C	109.5	N1—C13—C14	101.29 (10)
H1B—C1—H1C	109.5	C10—C13—C14	113.09 (11)
C1—C2—C3	113.51 (13)	N1—C13—H13	109.7
C1—C2—H2A	108.9	C10—C13—H13	109.7
C3—C2—H2A	108.9	C14—C13—H13	109.7
C1—C2—H2B	108.9	C15—C14—C13	102.50 (10)
C3—C2—H2B	108.9	C15—C14—H14A	111.3
H2A—C2—H2B	107.7	C13—C14—H14A	111.3
C4—C3—C2	113.33 (12)	C15—C14—H14B	111.3
C4—C3—H3A	108.9	C13—C14—H14B	111.3
C2—C3—H3A	108.9	H14A—C14—H14B	109.2
C4—C3—H3B	108.9	N2—C15—C16	120.89 (12)
C2—C3—H3B	108.9	N2—C15—C14	114.44 (11)
H3A—C3—H3B	107.7	C16—C15—C14	124.65 (11)
C3—C4—C5	114.86 (12)	C21—C16—C17	118.99 (12)
C3—C4—H4A	108.6	C21—C16—C15	120.56 (12)
C5—C4—H4A	108.6	C17—C16—C15	120.45 (12)
C3—C4—H4B	108.6	C18—C17—C16	120.23 (12)
C5—C4—H4B	108.6	C18—C17—H17	119.9
H4A—C4—H4B	107.5	C16—C17—H17	119.9
C6—C5—C4	112.80 (12)	C17—C18—C19	120.54 (13)
C6—C5—H5A	109.0	C17—C18—H18	119.7
C4—C5—H5A	109.0	C19—C18—H18	119.7
C6—C5—H5B	109.0	C20—C19—C18	119.61 (12)
C4—C5—H5B	109.0	C20—C19—H19	120.2
H5A—C5—H5B	107.8	C18—C19—H19	120.2
O1—C6—C5	107.04 (10)	C20—C21—C16	120.27 (13)
O1—C6—H6A	110.3	C20—C21—H21	119.9
C5—C6—H6A	110.3	C16—C21—H21	119.9
O1—C6—H6B	110.3	C19—C20—C21	120.37 (13)
C5—C6—H6B	110.3	C19—C20—H20	119.8
H6A—C6—H6B	108.6	C21—C20—H20	119.8
O1—C7—C8	124.77 (11)	O2—C22—N1	119.85 (13)
O1—C7—C12	115.21 (11)	O2—C22—C23	124.01 (12)
C8—C7—C12	120.00 (12)	N1—C22—C23	116.12 (12)
C7—C8—C9	119.52 (12)	C22—C23—H23A	109.5
C7—C8—H8	120.2	C22—C23—H23B	109.5
C9—C8—H8	120.2	H23A—C23—H23B	109.5
C10—C9—C8	121.11 (12)	C22—C23—H23C	109.5
C10—C9—H9	119.4	H23A—C23—H23C	109.5
C8—C9—H9	119.4	H23B—C23—H23C	109.5
C9—C10—C11	118.49 (12)		
			
C22—N1—N2—C15	175.04 (12)	C9—C10—C13—C14	−86.17 (15)
C13—N1—N2—C15	−1.30 (14)	C11—C10—C13—C14	91.00 (14)
C1—C2—C3—C4	−179.80 (13)	N1—C13—C14—C15	−0.22 (12)
C2—C3—C4—C5	−176.14 (12)	C10—C13—C14—C15	121.03 (11)
C3—C4—C5—C6	176.22 (11)	N1—N2—C15—C16	179.74 (11)
C7—O1—C6—C5	−170.59 (10)	N1—N2—C15—C14	1.13 (15)
C4—C5—C6—O1	70.78 (14)	C13—C14—C15—N2	−0.55 (14)
C6—O1—C7—C8	−0.01 (18)	C13—C14—C15—C16	−179.10 (11)
C6—O1—C7—C12	178.57 (11)	N2—C15—C16—C21	179.85 (12)
O1—C7—C8—C9	177.70 (12)	C14—C15—C16—C21	−1.68 (19)
C12—C7—C8—C9	−0.81 (19)	N2—C15—C16—C17	−0.64 (18)
C7—C8—C9—C10	−0.41 (19)	C14—C15—C16—C17	177.82 (12)
C8—C9—C10—C11	1.34 (19)	C21—C16—C17—C18	0.53 (19)
C8—C9—C10—C13	178.52 (12)	C15—C16—C17—C18	−178.98 (12)
C9—C10—C11—C12	−1.08 (19)	C16—C17—C18—C19	0.04 (19)
C13—C10—C11—C12	−178.36 (12)	C17—C18—C19—C20	−0.43 (19)
C10—C11—C12—C7	−0.12 (19)	C17—C16—C21—C20	−0.71 (19)
O1—C7—C12—C11	−177.58 (11)	C15—C16—C21—C20	178.80 (12)
C8—C7—C12—C11	1.07 (19)	C18—C19—C20—C21	0.2 (2)
C22—N1—C13—C10	63.40 (16)	C16—C21—C20—C19	0.3 (2)
N2—N1—C13—C10	−120.39 (12)	N2—N1—C22—O2	−172.82 (12)
C22—N1—C13—C14	−175.30 (12)	C13—N1—C22—O2	3.1 (2)
N2—N1—C13—C14	0.90 (13)	N2—N1—C22—C23	8.60 (18)
C9—C10—C13—N1	28.19 (17)	C13—N1—C22—C23	−175.47 (12)
C11—C10—C13—N1	−154.64 (11)		

### Hydrogen-bond geometry (Å, °)

**Table:**

*Cg1* and *Cg2* are the centroids of the C7–C12 and C16–C21 aromatic rings, respectively.

**Table:**

*D*—H···*A*	*D*—H	H···*A*	*D*···*A*	*D*—H···*A*
C8—H8···Cg2^i^	0.93	2.95	3.6252 (15)	131
C14—H14A···Cg2^ii^	0.97	2.63	3.5024 (14)	151
C19—H19···Cg1^iii^	0.93	2.71	3.4299 (15)	135

Symmetry codes: (i) *x*, −*y*−1/2, *z*−3/2; (ii) *x*+1, *y*, *z*; (iii) *x*−1, −*y*−1/2, *z*−1/2.
